# The effect of personalized orthopedic insoles on plantar pressure during running in subtle cavus foot

**DOI:** 10.3389/fbioe.2024.1343001

**Published:** 2024-02-22

**Authors:** Mujia Ma, Qingquan Song, Hui Liu

**Affiliations:** ^1^ School of Sport Science, Beijing Sport University, Beijing, China; ^2^ School of Strength and Conditioning Training, Beijing Sport University, Beijing, China; ^3^ China Institute of Sport and Health Science, Beijing Sport University, Beijing, China

**Keywords:** high-arched feet, foot pain, heel varus, forefoot valgus, Coleman block test

## Abstract

**Objective:** This study aims to investigate the patterns of plantar pressure distribution during running for patients with subtle cavus foot (SCF) and determine the impact of personalized orthopedic insoles with forefoot wedge on plantar pressure distribution in patients with SCF.

**Methods:** Sixteen undergraduate participants (8 with SCF and 8 with normal arches) were recruited based on arch height index measurements. Two full-length insoles were personalized for each SCF based on plantar pressures during running, an arch support insole (ASI) and an arch support with forefoot wedge insole (AFI). Foot pressure data collected during different insole conditions in running, analyzing ten regions of footprints for peak pressure and pressure-time integral.

**Results:** Higher peak pressures were observed in patients with SCF at the medial forefoot (*p* = 0.021), medial heel (*p* = 0.013), and lateral heel (*p* = 0.025), with a higher pressure-time integral also noted at the medial forefoot (*p* = 0.025), medial heel (*p* = 0.015), and lateral heel (*p* = 0.047) when compared to normal arches. Compared with without-insole, both the AFI and the ASI reduced peak pressure at the medial (AFI *p* = 0.011; ASI *p* = 0.024) and lateral heel (AFI *p* = 0.028; ASI *p* = 0.032). The AFI reduced peak pressure at the medial heel (*p* = 0.013) compared with the ASI. Both the AFI and the ASI reduced pressure-time integral at the medial forefoot (AFI *p* = 0.003; ASI *p* = 0.026), central forefoot (AFI *p* = 0.005; ASI *p* = 0.011), medial heel (AFI *p* = 0.017; ASI *p* = 0.005), and lateral heel (AFI *p* = 0.017; ASI *p* = 0.019). Additionally, the ASI reduced pressure-time integral at the big toe (*p* = 0.015) compared with the without-insole.

**Conclusion:** These findings demonstrate that during running in patients with SCF, plantar pressures are concentrated in the forefoot and heel compared to the normal arch. The personalized orthotic insoles can be used to effectively redistribute plantar pressure in patients with SCF running. Incorporating a forefoot wedge to specifically address the biomechanical abnormalities associated with SCF may enhance the effectiveness of orthopedic insoles.

## 1 Introduction

The subtle cavus foot (SCF) is characterized by a slight heel varus and a plantarflex firstray, however formal statistics on the incidence and prevalence of SCF are lacking (Deben and Pomeroy, 2014). Although the cavus foot is commonly caused by neurological disorders, SCF is considered to be a variant of normal as common as flatfoot deformities (Monoli and Graham, 2005). The Coleman block test detects if the heel varus for SCF is of primary origin or is secondarily caused by the plantarflexion of the first metatarsal (Deben and Pomeroy, 2014; Benjamin et al., 2020). A one-inch block is placed under the heel and lateral border of the foot. If the heel corrects to a normal or slightly valgus position, then the heel varus is forefoot-driven by a rigidly plantarflexed first ray. Failure of the heel varus to reduce indicates a rearfoot-driven condition.

The specific structure of patients with SCF may result in increased foot pressure, leading to frequent plantar pain during physical activity. Clinical support for this potential association has been largely extrapolated from studies examining the relationship between arch height and sports and overuse injuries, due to a lack of assessment of plantar pressure characteristics in patients with forefoot-driven SCF. In a static standing posture, individuals with high arches, as determined by arch index, showed a reduction in pressure distribution in the arch, accompanied by an increase in pressure distribution in both the forefoot and heel, compared to those with a normal arches (Woźniacka et al., 2019). During walking, the contact area is reduced, pressure (Carson et al., 2012; Woźniacka et al., 2019) and pressure-time integral (Burns et al., 2005) is higher in the heel and forefoot for those with high arches. Additionally, the center of pressure is lateral (Li, Xiang, and Zhang, 2020), and there is a notable reduction and delay in the initial peak force exerted by the big toe, compared to individuals with normal arches (Buldt et al., 2018). As a result, around 60% of individuals with high arches of either idiopathic or neurogenic aetiology experience fatigue while walking or running and often report oppressive pain in the heel and metatarsal heads, which has also been shown to correlate with high pressure-time integral (Burns et al., 2005). Over time, mechanical overloading can contribute to various conditions such as metatarsalgia, stress fractures, and other stress-related disorders of the ankle, knee, hip, and spine (Williams et al., 2001; Porter, 2018). This issue is compounded by activities like running, which increase foot loading and may further heighten the risk of pain and injuries (Hollander et al., 2019). Additionally, current studies of high-arched typically report collection of plantar pressure by means of a pressure plate placed on the ground (Fernández-Seguín et al., 2014; Woźniacka et al., 2019; Li, Xiang, and Zhang, 2020). In comparison, flexible pressure insoles provide a way to measure pressure between the plantar surface and the insole. With a method of assessing plantar pressure appropriate to the clinical setting, information about the plantar-insoles interface can inform insole prescriptions when concurrent foot symptoms are present. Consequently, understanding the distribution of plantar pressures at the plantar-insoles interface during running in patients with subtle cavus foot is crucial for guiding targeted interventions in pain management and injury prevention.

Most of the research on treating plantar pain in high-arched has focused on redistributing plantar pressure through the use of elastic insoles with shock-absorbing properties (Crosbie and Burns, 2007; Jung-Kyu et al., 2015). It has been shown that orthotic insoles for high-arched are effective in increasing contact area (Jung-Kyu et al., 2015) and distributing plantar pressure to reduce foot pain scores (Burns et al., 2006). In addition, it also reduces lower limb muscle activity, thereby increasing effective muscle utilization (Jung-Kyu et al., 2015). However, the Coleman block test confirms the flexible of the patients with SCF and the potential of foot orthoses to effectively realign the foot nature of the deformity (Manoli and Graham, 2018). Adjusting the foot to a neutral position can improve the biomechanical function of the foot, providing support and stability, and reducing discomfort or pain (Pascual et al., 2009). As a result, podiatrists recommend the use of a forefoot wedge pad and lowering the support under the arch to help correct SCF (Manoli and Graham, 2018; Benjamin et al., 2020). A study personalized full-length orthoses for SCF with ankle instability and pain. The orthosis was made of vinyl acetate with a groove under the first metatarsal head, a wedge on the lateral forefoot, a lowered arch, and a cushion on the heel. Follow-up questionnaires conducted after 1 and 2 years of orthotic use showed significant improvement in the patient’s pain and instability (LoPiccolo et al., 2010). However, further research is needed to understand the effects of forefoot wedge adjustments on dispersing plantar pressure in patients with SCF.

In summary, understanding plantar pressure distribution in patients with SCF and analyzing the effect of forefoot wedge on plantar pressure distribution are key factors in targeting pain management and injury prevention strategies in patients with SCF. Thus, this study aims to investigate the patterns of plantar pressure distribution during running for patients with SCF and determine the impact of orthopedic insoles with forefoot wedge on plantar pressure distribution in patients with SCF. According to the study of plantar pressure distribution in high-arch populations, we hypothesized that peak pressure and pressure-time integral are greater in the forefoot and heel during running in patients with SCF compared to those in normal arches. Furthermore, the arch support with forefoot wedge insoles is more effective at reducing plantar pressure on the forefoot and heel during running in patients with SCF compared to the arch support insole.

## 2 Methods

### 2.1 Participants

**FIGURE 1:**
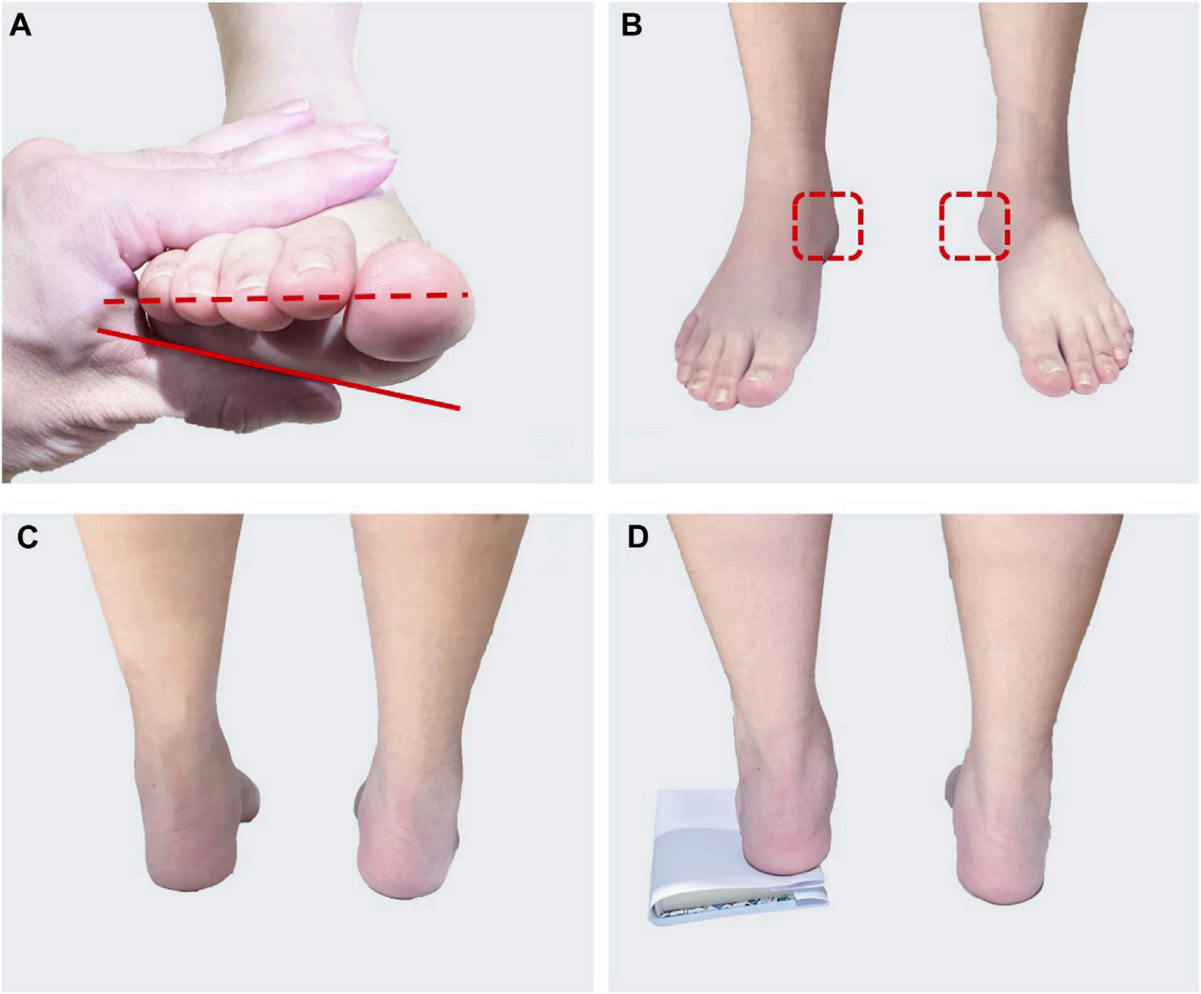
The patient with SCF characteristics: **(A)** Measuring forefoot valgus angle. The red solid line is the alignment of the first to fifth metatarsals head and the red dotted line is the horizontal line; **(B)** “peek-a-boo heel” sign, the red dashed box is the observed heel; **(C)** Standing position posterior view without block; **(D)** Coleman block test.

Thirty-five patients with potentially high arches were evaluated and 8 SCF patients (4 males and 4 females, age: 23.5 ± 2.7 years, height 1.72 ± 0.06 m, weight 65.1 ± 8.6 kg) were retained for testing. The patients with SCF had to satisfy the following criteria: high arches of unknown etiology and free of neurological disease, in the range of the Arch Height Index (AHI), forefoot valgus angle greater than 10°, with the sign of the “peek-a-boo heel,” a positive Coleman block test. AHI was measured as the vertical height of the dorsum at half of the total foot length divided by the truncated foot length (Williams and Mcclay, 2000). The SCF individuals were identified as those with AHI at least 1.5 standard deviations above the mean of a reference population (0.34 ± 0.03 arch height units), that is greater than 0.385 (Zifchock et al., 2006). The measurement of the forefoot valgus angle required the participant to be in the supine position, ensuring that the foot was in a neutral position. The angle between the alignment of the first to fifth metatarsals heads and the horizontal line was measured (Figure 1A) (Najjarine and Kielt, 2008). The “peek-a-boo heel” sign is determined from an anterior view of the foot, where a partial medial heel can be observed as the participant stands (Figure 1B) (Deben and Pomeroy, 2014). In the Coleman Block Test, a 1.5-cm rigid block of wood is placed under the heel and lateral aspect of the participant’s foot, and the sign is positive when the participant’s heel is corrected to normal valgus from a posterior view (Figures 1C, D) (Deben and Pomeroy, 2014).

Normal arch participants were matched based on gender, age, height, weight, foot length of the eight subjects in the SCF group and satisfied the AHI range of the reference population (4 males and 4 females, age: 23.5 ± 2.3 years, height 1.73 ± 0.08 m, weight 65.6 ± 6.9 kg). Anthropometric measures were similar between the groups, except the SCF group had a significantly higher AHI compared to the normal arch group (Table 1).

**TABLE 1:** Mean anthropometric measurements, including age, height, mass, foot length, and AHI, for both the SCF group and the normal arch group.

Group	Age (yr)	Height (m)	Mass (kg)	Foot length (cm)	AHI (unitless)
SCF (4 males, 4 females)	23.5 ± 2.7	1.72 ± 0.06	65.1 ± 8.6	24.0 ± 1.1	0.41 ± 0.03^a^
Normal Arch (4 males, 4 females)	23.3 ± 1.8	1.73 ± 0.08	65.6 ± 6.9	24.6 ± 1.5	0.33 ± 0.01^a^
*t*-value	0.060	0.146	0.128	0.940	6.751
*p*-value	0.953	0.886	0.900	0.363	<0.001

^a^ Significant difference (*p* < 0.05) between SCF, and the normal arches.

All sixteen participants were students with no professional athletic training experience at Beijing Sport University. Ethical approval for the study was obtained from the Sports Science Experiment Ethics Committee of Beijing Sport University (approval number 2020187H). All subjects were well informed about the study and allowed to ask questions before providing signed consent.

### 2.2 The execution procedure of personalized insoles

Two personalized full-length insoles, an arch support insole (ASI) and an arch support with forefoot wedge insole (AFI), were customized based on plantar pressure data collected using a Footscan plantar pressure collection system (RS Scan, Belgium) during barefoot running at normal speed for each SCF participant.

The design process, facilitated by Easy CAD (Easy CAD insole, Sensor Medica Sas, Italy), comprised five steps: 1) choosing a base insole template; 2) importing the individual’s plantar pressure data; 3) adjusting the thickness of the insole base and the height of the heel cup; 4) modifying the height of the arch and forefoot wedge; 5) smoothing the overall design. Following the customization of insole parameters according to the personalized design scheme, they were inputted into the CNC milling machine (CNC Vulcan Series, Sensor Medica Sas, Italy), the EVA blocks were carved and shaped, and finally the insoles were polished to match the lasts of the test shoes. The EVA block used to make the insoles is 30 mm thick with a hardness of 35 (Shore A) and has a 2 mm thick EVA base with a hardness of 60 (Shore A).

**FIGURE 2:**
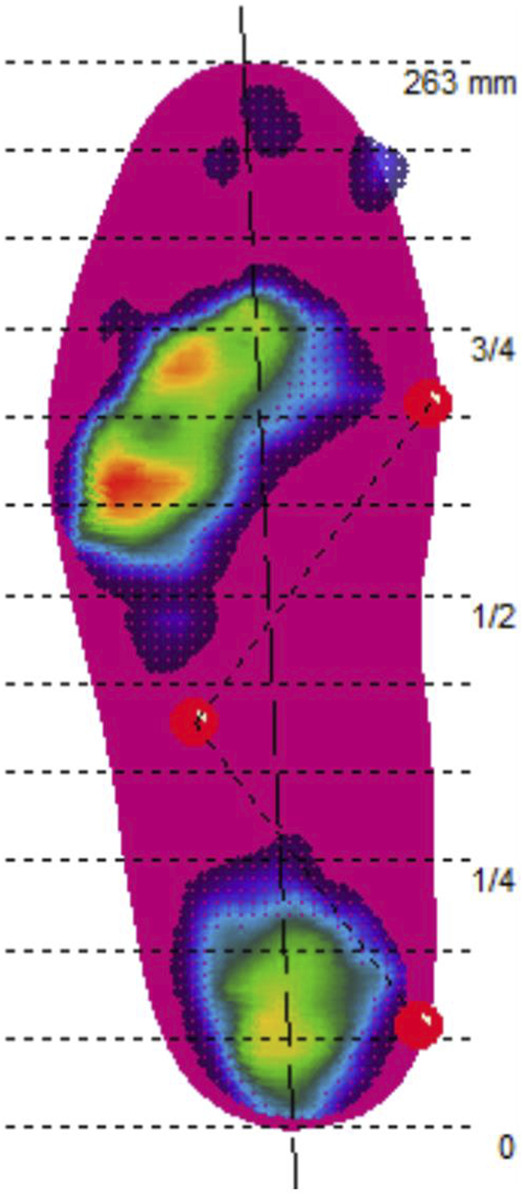
Three-point setup arch support structure range (Note: The horizontal dashed lines are the 13 isometric lines dividing the foot. The first point is located on the medial side of the foot, at the upper edge of the fifth isometric line; the second point is located on the lateral side of the foot, midway between the eighth and ninth isometric lines; and the third point is located on the medial side of the foot, at the upper edge of the twelfth isometric line).

**FIGURE 3:**
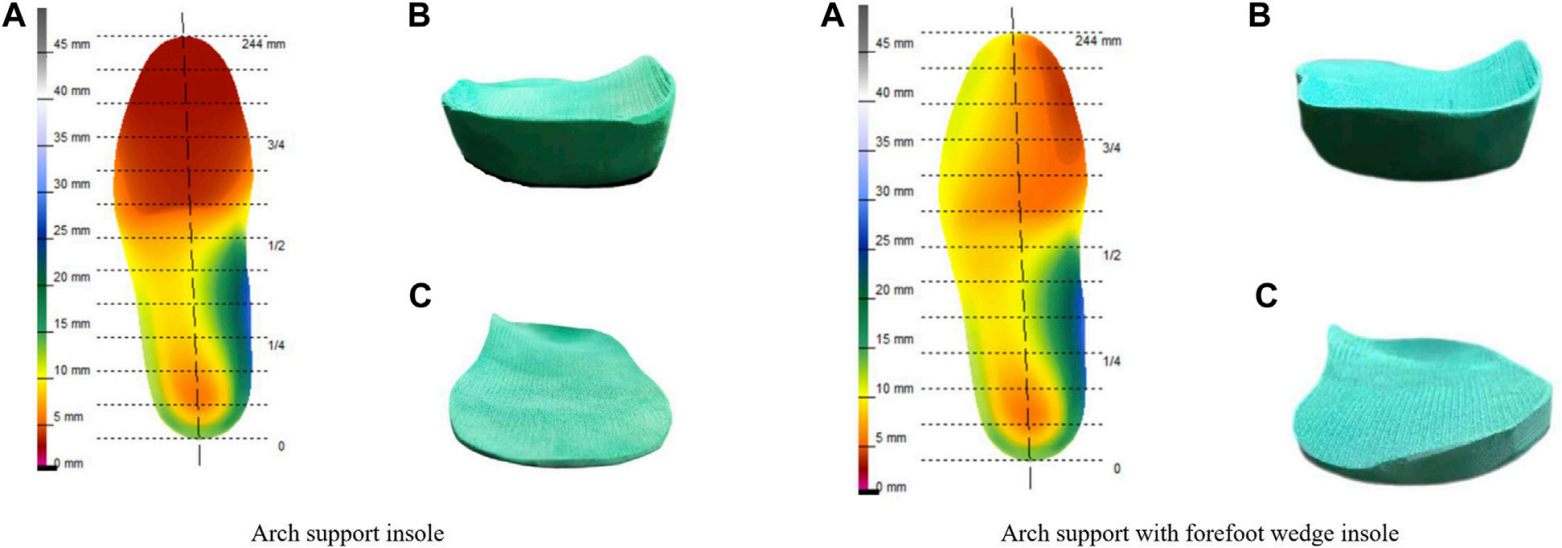
Arch support insole and arch support with forefoot wedge insole: **(A)** Design drawing; **(B)** The perspective of the heel position; **(C)** The perspective of the forefoot position.

The extent of the arch support structure is defined by three points and adjusted to the subject’s plantar pressure. The location of the three points that locate the arch support structure can be referenced to the 13 isometric lines that divide the foot. The first point is located on the medial side of the foot, at the upper edge of the fifth isometric line; the second point is located on the lateral side of the foot, midway between the eighth and ninth isometric lines; and the third point is located on the medial side of the foot, at the upper edge of the twelfth isometric line (Figure 2). The forefoot wedge starts on the outer side of the first metatarsal recess and continues all the way to the outer edge of the device. The arch support insole (ASI) featured arch support, while the arch support with the forefoot wedge insole (AFI) included both arch support and a forefoot wedge (Figure 3).

### 2.3 Experimental procedure

Plantar pressure data and running speed were recorded during running using the Pedar-X System (100 Hz, Novel Gmbh, Germany) and SmartSpeed (SmartSpeed, Australia), respectively. The normal arch participants were tested with without-insole. In contrast, the SCF participants were randomized to three different insole conditions: arch support insole, arch support with forefoot wedge insole, and without-insole. Before testing in each condition, subjects will run on a runway for 5 min to acclimatize to the insoles and after familiarization will be tested. There was a 15-min rest break between each test of the different conditions. All participants wore standardized socks and shoes (model S215, Maitan Inc., Zhejiang, China). The shoe is made with a polyurethane outsole and a stretch fabric upper.

Each participant completed three successful running trials on a 20-m runway under each condition. To standardize movement speeds, participants ran at a moderate speed (3.0 ± 0.3 m/s) (Lai et al., 2020).

### 2.4 Data analysis

As all subjects demonstrated right-leg dominance, the analysis focused on the right foot of each participant. To avoid the influence of acceleration or deceleration on plantar pressure measurements, the step where running speed had stabilized was selected for further analysis in each trial.

**FIGURE 4:**
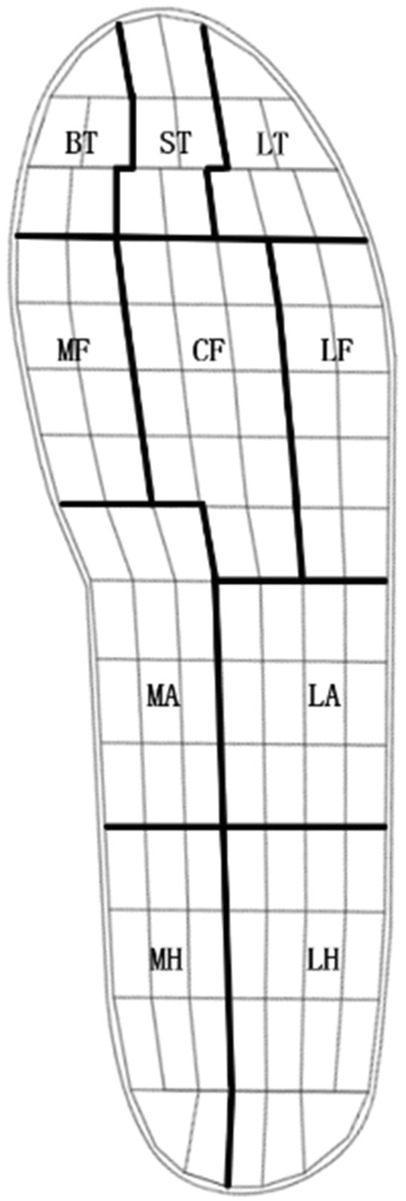
The footprints were divided into ten regions: big toe (BT), second toe (ST), lateral toes (LT), medial forefoot (MF), central forefoot (CF), lateral forefoot (LF), medial arch (MM), lateral arch (LM), medial heel (MH) and lateral heel (LH).

The footprints were divided into ten regions for analysis: big toe (BT), second toe (ST), lateral toes (LT), medial forefoot (MF), central forefoot (CF), lateral forefoot (LF), medial arch (MM), lateral arch (LM), medial heel (MH), lateral heel (LH) (Figure 4). The peak pressure (kPa) and pressure-time integral (kPa-s) were calculated for each region.

In this study, the pressure-time integral of the medial forefoot and lateral heel were considered the primary variables. This is based on the heel varus and excessive plantarflexion of the first metatarsal in patients with SCF, a feature that can lead to abnormal plantar pressure distribution. Moreover, reducing the pressure-time integral is a key strategy to reduce foot pain.

### 2.5 Statistical analysis

Statistical analyses were performed using the SPSS 27.0 software package. Prior to statistical analysis, a normality test was conducted on all variables. For data conforming to normal distribution, independent samples t-tests were used to compare anthropometric measurements, as well as peak pressure and pressure-time integral for each foot region between the SCF and normal arch groups. For normally distributed data, one-way repeated measures ANOVA was performed, followed by a *post hoc* test corrected for multiple comparisons using LSD, to identify the effects of the three insole conditions in patients with SCF. For data that did not meet the normality assumption, the Mann-Whitney *U* test (for two independent samples) and the Friedman test (for repeated measures data) were employed. The significance level was set at *p* < 0.05 in all analyses.

## 3 Results

Anthropometric measurements were similar between SCF and the normal arches (Table 1). The SCF group and the normal arches group were similar in age, height, body mass, and foot length. AHI values were significantly greater in patients with SCF compared to normal arches (*p* < 0.001).

Peak pressures during running in patients with SCF were greater in the medial forefoot (*p* = 0.021), lateral forefoot (*p* = 0.025), medial heel (*p* = 0.013), and lateral heel (*p* = 0.025) than in those with normal arches, whereas peak pressures at the medial arch were smaller than normal arches (*p* = 0.045) (Table 2). Pressure-time integral during running in patients with SCF was greater in the medial forefoot (*p* = 0.025), medial heel (*p* = 0.015), and lateral heel (*p* = 0.047) than in those with normal arches (Table 2). There were no other significant differences in peak pressures or pressure-time integral was observed between the two groups (*p* > 0.05).

**TABLE 2:** Comparing peak pressure (kpa) and pressure-time integral (kpa·s) characteristics between the SCF group and the normal arches group.

Foot region	Peak pressures				Pressure-time integral			
	SCF	Normal arches	*t*-value	*p*-value	SCF	Normal arches	*t*-value	*p*-value
Big toe	309.6 ± 90.7	307.4 ± 97.6	0.048	0.963	41.5 ± 12.1	42.9 ± 10.6	−0.235	0.818
Second toe	241.2 ± 149.1	197.3 ± 73.6	0.746	0.468	34.3 ± 17.0	29.1 ± 8.4	0.771	0.454
Lateral toe	188.0 ± 68.6	165.4 ± 45.0	0.779	0.449	26.3 ± 9.8	25.3 ± 8.2	0.222	0.827
Medial forefoot	367.5 ± 53.9^a^	294.1 ± 58.5^a^	2.609	0.021	51.8 ± 9.0^a^	41.9 ± 6.5^a^	2.517	0.025
Central forefoot	464.5 ± 73.9	401.0 ± 69.6	1.771	0.098	63.8 ± 9.8	57.3 ± 17.1	0.928	0.369
Lateral forefoot	233.6 ± 55.5^a^	180.1 ± 24.0^a^	2.503	0.025	31.6 ± 9.5	26.0 ± 4.3	1.525	0.149
Medial arch	70.5 ± 17.5^a^	89.8 ± 17.6^a^	−2.196	0.045	6.1 ± 1.5	6.6 ± 2.4	−0.521	0.611
Lateral arch	111.6 ± 30.1	126.7 ± 32.9	−0.953	0.357	11.0 ± 3.5	13.6 ± 3.6	−1.487	0.159
Medial heel	429.3 ± 120.3^a^	289.9 ± 37.3^a^	3.130	0.013	34.8 ± 5.6^a^	28.0 ± 3.9^a^	2.784	0.015
Lateral heel	457.9 ± 150.0^a^	304.5 ± 64.4^a^	2.657	0.025	37.3 ± 9.9^a^	28.3 ± 6.3^a^	2.176	0.047

^a^ Significant difference (*p* < 0.05) between SCF, and the normal arches.

There were differences in peak pressure among different insole conditions for SCF in each of the four defined foot regions (Table 3). Peak pressures were significantly different between the medial arch (*p* < 0.001), lateral arch (*p* = 0.048), medial heel (*p* = 0.002), and lateral heel (*p* = 0.023). Post hoc analyses indicated that compared to without-insole, both the AFI and the ASI significantly reduced peak pressures in the medial (AFI *p* = 0.011; ASI *p* = 0.024) and lateral heels (AFI *p* = 0.028; ASI *p* = 0.032). Specifically, the AFI also significantly reduced peak pressure in the medial heel (*p* = 0.013) when compared to the ASI. Conversely, compared to the without-insole condition, both the AFI (*p* = 0.003) and ASI (*p* < 0.001) resulted in increased peak pressure in the medial arch, and the ASI alone increased peak pressure in the lateral arch (*p* = 0.048). There were no significant differences in peak pressures in the big toe, second toe, lateral toe, medial forefoot, central forefoot, and lateral forefoot across the different conditions (*p* > 0.05).

**TABLE 3:** Comparing peak pressure (kpa) in the SCF across different insole conditions.

Foot region	Without-insole	Arch support with forefoot wedge insole	Arch support insole	*F*-value	*p*-value
Big toe	309.6 ± 90.7	279.8 ± 70.7	253.5 ± 102.3	3.080	0.078
Second toe	241.2 ± 149.1	258.5 ± 83.2	222.7 ± 98.2	0.315	0.734
Lateral toe	188.0 ± 68.6	223.9 ± 90.6	198.0 ± 108.6	0.422	0.664
Medial forefoot	367.5 ± 53.9	329.6 ± 83.0	342.8 ± 107.4	0.913	0.424
Central forefoot	464.5 ± 73.9	385.6 ± 100.0	418.1 ± 79.4	2.067	0.163
Lateral forefoot	233.6 ± 55.5	215.7 ± 71.4	236.6 ± 92.5	0.910	0.425
Medial arch	70.5 ± 17.5^a^ ^b^	125.8 ± 28.7^a^	118.1 ± 15.1^b^	19.806	<0.001
Lateral arch	111.6 ± 30.1^b^	128.7 ± 24.1	143.5 ± 33.8^b^	3.793	0.048
Medial heel	429.3 ± 120.3^a^ ^b^	282.6 ± 121.7^a^ ^c^	316.1 ± 120.0^b^ ^c^	10.118	0.002
Lateral heel	457.9 ± 150.0^a^ ^b^	324.0 ± 128.3^a^	324.4 ± 112.1^b^	4.984	0.023

^a^ Significant difference (*p* < 0.05) between the without-insole and the arch support with forefoot wedge insole.

^b^ Significant difference (*p* < 0.05) between the without-insole and the arch support insole.

^c^ Significant difference (*p* < 0.05) between the arch support with forefoot wedge insole and the arch support insole.

There were differences in pressure-time integral among different insole conditions for SCF in each of the seven defined foot regions (Table 4). Pressure-time integral were significantly different between the big toe (*p* = 0.044), medial forefoot (*p* = 0.024), central forefoot (*p* = 0.001), medial arch (*p* < 0.001), lateral arch (*p* = 0.013) and medial heel (*p* = 0.003), lateral heel (*p* = 0.010). Post hoc analyses indicated that compared to without-insole, both the AFI and the ASI significantly reduced the pressure-time integral at the medial forefoot (AFI *p* = 0.003; ASI *p* = 0.026), central forefoot (AFI *p* = 0.005; ASI *p* = 0.011), medial heel (AFI *p* = 0.017; ASI *p* = 0.005), and lateral heel (AFI *p* = 0.017; ASI *p* = 0.019). Additionally, the ASI alone significantly reduced the pressure-time integral at the big toe (*p* = 0.015). However, comparison to the without-insole, both the AF and ASI increased the pressure-time integral at the medial arch (AFI and ASI *p* < 0.001) and lateral arch (AFI *p* = 0.008; ASI *p* = 0.019). There were no significant differences in pressure-time integral in the second toe, lateral toe and lateral forefoot across the different conditions (*p* > 0.05).

**TABLE 4:** Comparing pressure-time integral (kpa·s) in the SCF across different insole conditions.

Foot region	Without-insole	Arch support with forefoot wedge insole	Arch support insole	*F*-value	*p*-value
Big toe	41.5 ± 12.1^a^	35.3 ± 8.6	32.3 ± 14.8^a^	3.929	0.044
Second toe	34.3 ± 17.0	34.2 ± 11.6	29.5 ± 13.5	0.424	0.662
Lateral toe	26.3 ± 9.8	31.4 ± 14.0	25.5 ± 14.9	0.684	0.520
Medial forefoot	51.8 ± 9.0^a^ ^b^	40.1 ± 8.6^b^	40.9 ± 12.1^a^	4.902	0.024
Central forefoot	63.8 ± 9.8^a^ ^b^	48.4 ± 11.2^b^	51.1 ± 8.1^a^	11.271	0.001
Lateral forefoot	31.6 ± 9.5	29.6 ± 12.3	31.0 ± 13.7	0.698	0.514
Medial arch	6.1 ± 1.5^a^ ^b^	14.0 ± 3.4^b^	12.7 ± 2.4^a^	32.564	<0.001
Lateral arch	11.0 ± 3.5^a^ ^b^	14.3 ± 3.5^b^	16.0 ± 5.2^a^	5.989	0.013
Medial heel	34.8 ± 5.6^a^ ^b^	25.2 ± 8.1^b^	26.0 ± 8.1^a^	9.023	0.003
Lateral heel	37.3 ± 9.9^a^ ^b^	25.9 ± 6.9^b^	28.9 ± 11.4^a^	6.457	0.010

^a^ Significant difference (*p* < 0.05) between the without-insole and the arch support insole.

^b^ Significant difference (*p* < 0.05) between the without-insole and the arch support with forefoot wedge insole.

## 4 Discussion

This study aims to examine the patterns of plantar pressure distribution while running for patients with SCF and assess the effects of using arch support insoles and arch support insoles with forefoot wedges on plantar pressure distribution. The major findings of this study demonstrate that the peak pressure and pressure-time integral were higher in the forefoot and heel during running in patients with SCF compared to the normal arches. In addition, both the arch support insole and the arch support with forefoot wedge insole significantly reduced forefoot and heel pressure-time integral and heel peak pressure during running in patients with SCF, but the arch support with forefoot wedge insole was more effective in reducing medial heel peak pressure. The results support the research hypothesis.

Running in patients with SCF was characterized by greater pressure–time integrals in the medial forefoot and medial and lateral heel. This is consistent with the previously reported most common areas of pain in people with high arches, which are the metatarsal and heel (Burns et al., 2005). It has also been reported that a medial plantar callus often forms below the first metatarsophalangeal joint in patients with SCF (Mann and Mann, 2004). Evidence suggests that regardless of etiology, cavus foot is characterized by abnormally high pressure–time integrals which are significantly related to foot pain (Burns et al., 2005). Although the patients with SCF lateral forefoot did not exhibit a greater pressure-time integral, higher peak pressures also contributed to the higher rate of lateral metatarsal injuries in people with high arches (Manoli and Graham, 2005). Variation in pressure is associated with changes in the moments acting on the proximal joints of the foot, which alter the pressure exerted on the tissues affecting the joints (Mohamud, 2020). As a result, high arch feet is associated with a higher rate of lower extremity injuries, most of which are skeletal, compared to normal and flat feet (Kaufman et al., 1999; Williams et al., 2001).

Excessive plantarflexion of the first metatarsal, excessive calcaneal pitch, and the lack of weight bearing under the arch are the main causes of plantar pain and stress injury in patients with SCF. The primary deformity in patients with SCF is plantarflexion of the first metatarsal, which is caused by overactivity of the peroneus longus, resulting in a flexible heel with limited valgus (Manoli and Graham, 2018). This plantarflexed position results in the first metatarsal head contacting the ground before the heel full valgus. The first ray then acts as a kickstand and abbreviates the flexible phase of the gait cycle. Specifically, the plantarflexed first ray tips the plane so that the heel is in varus with reduced cushioning and increased lower extremity impact forces (Williams et al., 2001). The larger calcaneal pitch in people with high arches increases heel pressure and less arch pressure during running. The calcaneal pitch angle is the angle between the line connecting the lowest two points on the lower surface of the heel bone and the horizontal plane. The sagittal plane angle of inclination of the heel bone is 10°–25° in people with normal arches and greater than 30° in people with cavus feet (Benjamin et al., 2020). It has been shown that calcaneal pitch is positively correlated with peak heel pressure and negatively correlated with arch pressure. In addition, people with high arches runs with less displacement in the internal and external directions of the center of pressure (Cock et al., 2008) and a faster rate of displacement in the anterior direction (Li et al., 2020). Unable to distribute the load through arch support, it is easy to transfer the load from the heel to the forefoot, which increases the forefoot pressure. When running is viewed as a cyclical loading event, these factors may lead to less shock absorption at heel strike, eccentric bony loads, and possible attenuation of the lateral soft-tissue structures. The foot has increased rigidity, decreased energy dissipation, and is prone to foot pain and stress injuries (Manoli and Graham, 2005; Deben and Pomeroy, 2014).

The results of the study showed that wearing orthotic insoles for patients with SCF significantly reduced the pressure-time integral to the forefoot and heel, consistent with previous studies. Burns demonstrated that the use of custom-made foot orthoses led to a 26% decrease in pressure-time integral and a corresponding 74% decrease in foot pain among a group of 154 individuals with cavus feet from different causes (Burns et al., 2006). The patients with SCF did not exhibit significant variations in peak pressure at the medial and central forefoot, but the pressure-time integral at the areas was significantly lower and peak pressures and pressure-time integral were significantly higher at the medial arch. This suggests that the decrease in the medial and central forefoot pressure-time integral is the result of an increase in arch contact time and a decrease in forefoot contact time. Wearing orthotic insoles increases the arch landing area (Jung-Kyu et al., 2015) and therefore increases arch support time and decreases the speed of the forward center of pressure movement. Reduced forefoot contact time during running in patients with SCF reduces pressure-time integral and effectively relieves forefoot pain. The decrease in heel peak pressure and pressure-time integral may be related to changes in the calcaneal pitch. It has been shown that high archers have a reduced calcaneal pitch when standing in semi-customized insoles with arch support (Eslami et al., 2009). In addition, the heel cup design reduces impact and increases ankle stability during running (Manoli and Graham, 2005; Yang et al., 2022). These findings suggest that personalized orthotic insoles are effective in distributing plantar pressure during running in patients with SCF.

However, there are also differences in the effects of the AFI and the ASI on plantar pressures in patients with SCF. The patients with SCF wearing arch support insoles showed an increase in peak pressure at the lateral arch and a decrease in the pressure-time integral at the big toe, compared to without-insole. In addition, peak pressures were higher at the medial heel, compared to AFI. Some studies suggest that the forefoot-driven SCF results in heel varus because of forefoot valgus, and shoes or orthotics that elevate the arch only cause the foot to varus further (Deben and Pomeroy, 2014). Therefore, an orthotic insole with only added arch support may result in patients with SCF running with a more lateral center of pressure. This increases lateral arch pressure, decreases big toe contact time to reduce the pressure-time integral. There was no significant difference in the medial heel pressure-time integral in SCF patients when wearing the two types of insoles. However, the reduction in medial heel peak pressure was more significant with AFI. This outcome could be due to an increase in medial heel strike time. It also suggests that arch support alone might cause the center of pressure to shift laterally in SCF patients, thereby reducing the time the medial side of the foot spends on the ground. As a result, to improve the efficacy of orthotic insoles, it is important to add a forefoot wedge to address the biomechanical abnormality in patients with SCF.

## 5 Conclusion

During running in patients with SCF, plantar pressures are concentrated in the forefoot and heel compared to the normal arch. The personalized orthotic insoles can be used to effectively redistribute plantar pressure in patients with SCF running. Incorporating a forefoot wedge to specifically address the biomechanical abnormalities associated with SCF may enhance the effectiveness of orthopedic insoles.

## 6 Study limitations

There are several limitations in this study. Firstly, this study focused on patients with SCF, and participants were screened more rigorously than in previous studies that have focused on high arch populations. This meticulous screening process resulted in all participants fitting the SCF profile despite the relatively small sample size. As a result, the present study provides detailed results on two key variables, medial forefoot and lateral heel pressure impulse, and these results provide an important reference for future research. With the data, future studies can more accurately calculate the required sample sizes with a view to obtaining more powerful results. Second, all participants were undergraduates with no experience of professional athletic training, so caution is needed when extrapolating these results to populations with different training backgrounds and age groups. Additionally, only one hardness of insole was used in this study, and the use of insoles with different hardnesses may produce different cushioning effects. Lastly, this study was cross-sectional, considering only the immediate effects of the insoles. The long-term stability of cushioning is also crucial for the protective role of insoles. Future research should prospectively evaluate the effectiveness of different personalized orthotic insoles in preventing foot pain and lower limb injuries in SCF patients during endurance activities.

## Data Availability

The datasets presented in this article are not readily available because Ethical restrictions were placed upon this data. Requests to access the datasets should be directed to MM, mamujia5683@gmail.com.
